# Loneliness, Stress, and Depressive Symptoms Among Older Immigrants: A Moderating Role of Familial Relationships

**DOI:** 10.1093/geroni/igab046.466

**Published:** 2021-12-17

**Authors:** Heejung Jang

**Affiliations:** University of Michigan, Ann Arbor, Michigan, United States

## Abstract

Objectives: Immigration is a stressful life event, and immigrants commonly experience loneliness, a risk factor for depression. However, little is known about how and whether older immigrants’ perceived stress exposure/appraisals mediate the association between loneliness and depressive symptoms. Further, this study explores whether familial relationships moderate the indirect or direct effects of the mediation models. Method: This study uses the 2012 Health and Retirement Study from a sample of 719 immigrants age 57 and older. A series of moderated mediation analyses were conducted across the total number of stress exposure and eight stress appraisal domains. Results: The findings indicate that the total number of stress exposure and five domains of stress appraisals (health problems in self, physical/emotional problems in spouse/child, financial strain, housing problems, and close relationships in others) mediate the association between loneliness and depressive symptoms. In addition, the perceived negative strain from family moderated the mediating effect of health problems and housing problems in the relationship between loneliness and depressive symptoms. Discussion: This study suggests that negative relationships with family may increase upsetting in stress appraisals on health and housing problems, which turn in increased depressive symptoms for lonely older immigrants. Practitioners need to assess older immigrants’ stressors and family relationships to understand their loneliness and depressive symptoms.

